# Development of Polylactic Acid–Curcumin Composite Films with Dual-Metal-Doped Copper Oxide Nanoparticles for Sustainable Antioxidant, Biocompatible, Photothermal, and Antibacterial Performance

**DOI:** 10.3390/polym18131626

**Published:** 2026-06-30

**Authors:** Gopinath Kasi, Sarinthip Thanakkasaranee, Nattan Stalin, Tae-Sik Park, Ramar Dharmaraj, Kittisak Jantanasakulwong, Nuttapol Tanadchangsaeng, Pornchai Rachtanapun

**Affiliations:** 1 Division of Packaging Technology, Faculty of Agro-Industry, Chiang Mai University, Chiang Mai 50100, Thailand; gopiscientist@gmail.com (G.K.); sarinthip.t@cmu.ac.th (S.T.); kittisak.jan@cmu.ac.th (K.J.); 2 Center of Excellence in Agro Bio-Circular-Green Industry (Agro BCG), Chiang Mai University, Chiang Mai 50100, Thailand; 3 Center of Excellence in Materials Science and Technology, Chiang Mai University, Chiang Mai 50200, Thailand; 4 Department of Life Science, Gachon University, Seongnam 13120, Republic of Korea; nattanstalin@gmail.com (N.S.); tspark@gachon.ac.kr (T.-S.P.); 5 Department of Botany, Alagappa University, Karaikudi 630003, Tamil Nadu, India; dharmarajramar1025@gmail.com; 6 College of Biomedical Engineering, Rangsit University, Lak-Hok, Pathumthani 12000, Thailand; nuttapol.t@rsu.ac.th

**Keywords:** active food packaging, biocompatibility, copper oxide nanoparticles, curcumin, nucleating agents, polylactic acid, sustainable material innovation, synergistic effects

## Abstract

Polylactic acid (PLA)-curcumin (CCM) composites, incorporating various contents of surface-functionalized dual-metal-doped copper oxide (SF-M-CuO), were prepared by the solution casting method. Synthesized composite films were evaluated for their antioxidant, biocompatible, photothermal, and antibacterial properties. The 4% CCM exhibits excellent compatibility based on total color difference, antioxidant activity, and controlled curcumin release behavior. In addition, different contents of SF-M-CuO (1–4%) were added to the PLA-4%-CCM polymer matrix. Synthesized composite films were characterized through functional, structural, and topographical analyses. FTIR and XRD analyses confirmed the successful incorporation of CCM and SF-M-CuO into the PLA matrix, which enhanced interfacial interactions and increased the crystallinity index by acting as effective nucleating agents. ABTS and DPPH radical scavenging assays revealed dose-dependent antioxidant activity due to the synergistic effects of CCM and SF-M-CuO. Biocompatibility evaluation using RAW 264.7 macrophage cells demonstrated non-toxic responses and enhanced cell proliferation in PLA-4%-CCM composite films containing up to 3%-SF-M-CuO. Among the fabricated films, PLA-4%-CCM-3%-SF-M-CuO exhibited superior photothermal performance and excellent antibacterial activity against *Staphylococcus aureus* and *Escherichia coli*, reducing bacterial counts to below the limit of detection. These findings demonstrate the potential of PLA-4%-CCM-3%-SF-M-CuO composite films as sustainable multifunctional materials for food safety and biomedical applications.

## 1. Introduction

Foodborne diseases cause serious health problems. These diseases affect about 600 million people and cause approximately 420,000 deaths each year [1]. The World Health Organization aims to ensure universal access to safe and healthy food by 2030 [2]. The food industry prioritizes product safety and control of microbial spoilage [3]. Conventional preservatives and antibiotics control microbes. By contrast, antibiotics lose effectiveness due to thermal instability and increasing antibiotic resistance [4,5,6]. Therefore, researchers explore inorganic nanoparticles (NPs), especially metal oxides such as calcium oxide (CaO) [7], zinc oxide (ZnO) [8,9], and copper oxide (CuO) [9,10]. These materials show strong amicrobial and photocatalytic activity and support in antibacterial packaging, and biomedical applications [7,8,9,10].

Active packaging systems release or absorb active compounds to maintain food quality and extend shelf life [11]. These systems interact with packaged food and the surrounding environment to enhance food safety during storage [12]. In contrast, conventional packaging has several limitations, including environmental pollution, high energy consumption during production, and potential risks associated with chemical migration [13,14]. Compared to conventional packaging, active packaging reduces food spoilage, inhibits microbial growth, minimizes food waste, and supports sustainable packaging practices [15]. Therefore, the development of biodegradable packaging materials with strong antibacterial properties is highly desirable for sustainable food-packaging applications [16].

Polylactic acid (PLA) is a semicrystalline biodegradable polymer derived from plant sources such as maize, sugarcane, potato, and cassava starch, and it serves as an alternative to petroleum-based plastics [17,18]. The U.S. Food and Drug Administration recognizes PLA as non-toxic and safe for food contact [19]. PLA provides good strength and processability for sustainable food packaging [17]. However, PLA has poor antioxidant and antibacterial properties, and high UV transmittances limit long-term use [20], so researchers add bioactive compounds and functional NPs to improve antibacterial and antioxidant performance.

Curcumin (CCM) is a hydrophobic bioactive compound obtained from *Curcuma longa* rhizome. It exhibited antioxidant, anti-inflammatory, antimicrobial, antitumor, and wound-healing activities and maintains high biocompatibility [21]. The European Food Safety Authority defines the acceptable daily intake of CCM as 0–3 mg/Kg of body weight [22]. CCM is incorporated into biodegradable polymers for food packaging to improve food safety and shelf life and to enable to active and intelligent packing due to its natural antioxidant [23,24], antibacterial [23,24], and pH-sensitive properties [25]. The incorporation of CCM increases the crystallinity of PLA films by acting as a nucleating agent and promoting physical interactions between PLA and CCM. In addition, its uniform dispersion within the polymer matrix enables controlled release, thereby overcoming poor solubility and instability while ensuring sustained antibacterial and antioxidant performance in active packaging systems [23]. Roy and Rhim reported that PLA-CCM films inhibited *Escherichia coli* (*E. coli*) and *Listeria monocytogenes* (*L. monocytogenes*) by inhibiting the growth of 1–2 log units. The slow release of CCM from the polymer matrix causes this antibacterial effect. By contrast, the films showed limited overall efficacy [23]. Rathod et al. evaluated the cytotoxicity of CCM on RAW 264.7 cells and reported on the IC_50_ value of 0.029 mg/mL [26]. Therefore, researchers need alternative strategies, such as adding functional nanofillers to enhance the antibacterial performance of PLA-CCM composite films.

The CuO NPs are p-type semiconductors with a narrow band gap of approximately 1.2 eV. CuO NPs exhibit excellent electrical, catalytic, and optical properties [27]. These properties make CuO NPs attractive for active food packaging applications. CuO NPs provide broad-spectrum antibacterial activity, low cost, good chemical stability, and ease of large-scale production [28]. Therefore, researchers have modified CuO NPs to improve their functional performance. Researchers widely employ metal-ion doping to tailor the physicochemical and biological properties of CuO [27,28,29]. Silver (Ag) doping enhances antibacterial activity by increasing reactive oxygen species (ROS) generation and disrupting bacterial cell membranes. Magnesium (Mg) doping improves antibacterial activity, biocompatibility, and surface reactivity [29]. The combined incorporation of Ag and Mg into CuO NPs is expected to provide synergistic effects, resulting in improved antibacterial and biocompatible properties compared with pure CuO NPs [29].

Researchers have widely studied nanomaterials and metal oxides, especially silver (Ag) and ZnO NPs, as fillers in PLA matrix to improve antibacterial activity, barrier properties and mechanical strength [23,30,31]. However, direct incorporation of unmodified NPs often leads to poor dispersion, aggregation, and weak interfacial compatibility with polymers. Surface functionalization with silane coupling agents offers a solution, improving nanoparticle dispersion, interfacial adhesion, and the overall performance of nanocomposites [32,33,34]. However, limited research has been reported on surface functionalization using silane coupling agents. Dowan Kim et al. reported that surface-modified tetrapod ZnO whiskers, functionalized with 3-(trimethoxysilyl)propyl methacrylate (KH570) and incorporated into poly(urethane acrylate) composite films, exhibited enhanced barrier and antibacterial properties [32]. Furthermore, the (3-glycidyloxypropyl)trimethoxysilane (GPTMS) is a bifunctional silane that enhances interfacial bonding and dispersion between organic polymers and inorganic nanomaterials [35]. Heydari et al. reported that covalent binding of chitosan (CS) with ZnO NPs via GPTMS improved the biocompatibility of nanomaterials at a concentration of 50 µg/mL in L929 fibroblast cells [36]. Furthermore, GPTMS has been used as a surface modifier for NPs to enhance antibacterial activity [37,38,39]. For biomedical applications, PLA-based nanocomposites provide biocompatibility, controlled drug release, and antibacterial properties, which are important for healthcare applications [18]. Researchers frequently used carbon-based materials such as multi-walled carbon nanotubes (MWCNTs) [40]. The MWCNTs embedded in PLA nanofibers increase local temperature under near-infrared irradiation, which leads to enabling localized hyperthermia [40]. In this context. The present study incorporates the bioactive compound CCM and dual metal-doped CuO into a PLA polymer matrix to form composite films and evaluates their photothermal response.

Previous work by our research team investigated Ag- and Mg-doped CuO (Ag:Mg:CuO = 3:3:94) NPs, which exhibited enhanced antibacterial activity and good biocompatibility [29]. On the next move, these NPs were further functionalized with GPTMS to improve dispersion and interfacial compatibility. Building on this, the present study incorporates surface-functionalized dual metal-doped CuO (SF-M-CuO) into a CCM-loaded PLA matrix. To the best of current knowledge, no study has yet reported such an integration to enhance antioxidant, antibacterial, photothermal, and biocompatible properties simultaneously. In this work, bioactive PLA-based composite films were fabricated via solution casting and systematically evaluated for their structural, functional, antioxidant, biocompatibility, photothermal and antibacterial properties to establish their potential for active food packaging and biomedical applications.

## 2. Materials and Methods

### 2.1. Chemicals

Polylactic acid (PLA, grade 4043D; Mw ≈ 111,000 g/mol) pellets were sourced from Precision Resource Co., Ltd., Mueang District, Nonthaburi, Thailand. Curcumin (CCM) (CAS-No: 458-37-7), (3-glycidyloxypropyl)-trimethoxysilane (GPTMS) (CAS No: 2530-83-8), and poly(ethylene glycol) [PEG: Mn. 8000] (CAS-No: 25322-68-3) were obtained from Sigma-Aldrich, St. Louis, MO, USA. Chloroform (CHCl_3_) (CAS-No: 67-66-3), silver nitrate [AgNO_3_] (CAS No: 7761-88-8) and Sodium hydroxide (NaOH) (CAS-No: 1310-73-2) were purchased from RCI Labscan, Bangkok, Thailand. Copper(II) nitrate trihydrate [Cu(NO_3_)_2_·3H_2_O] (CAS-No: 10031-43-3) was supplied by QreC, New Zealand, while magnesium nitrate hexahydrate [Mg(NO_3_)_3_·6H_2_O] (CAS-No: 13446-18-9) was obtained from Kemaus, Australia. All biological media were purchased from TM Media, Delhi, India. Deionized (DI) water was used throughout the experiment.

### 2.2. Characterization

#### 2.2.1. Crystal Structure

The X-ray diffraction (XRD) analysis was carried out on the synthesized powder of dual metal-doped CuO NPs, curcumin and composite films at 25 °C using an XPERT-PRO diffractometer (PANalytical, Almelo, The Netherlands) with Ni-filtered Cu Kα_1_ radiation (λ = 1.5406 Å). Measurements were collected over a 2θ range of 10–80° at 40 kV and 30 mA with a continuous scan and a step size of 0.05°.

#### 2.2.2. Surface Chemistry

X-ray photoelectron spectroscopy (XPS) analysis of dual-metal-doped CuO NPs was carried out using a PHI VersaProbe III spectrometer (Physical Electronics, Chanhassen, MN, USA) with monochromatic Al Kα radiation.

#### 2.2.3. Particle Size and Crystallinity

High-resolution transmission electron microscopy (HR-TEM), together with selected area electron diffraction (SAED) analyses were carried out on a Tecnai G2 20 S-TWIN microscope (FEI Company, Hillsboro, OR, USA) at 200 kV.

#### 2.2.4. Surface Morphology and Elemental Composition

Surface morphology was examined by scanning electron microscopy (SEM) using a JSM-IT200 system (JEOL Ltd., Tokyo, Japan) operated at an accelerating voltage of 15 kV. Before analysis, the composite film samples were coated with a thin layer of gold using a DII-29030SCTR smart coater to enhance conductivity before imaging, with the sputtering process lasting for 30 s. Elemental mapping and energy-dispersive X-ray (EDX) spectroscopy were utilized to identify the elemental composition the composite films.

#### 2.2.5. Chemical Structure

Fourier transform infrared (FTIR) analysis was performed using a Bruker Alpha II instrument (Bruker Optik GmbH, Ettlingen, Baden-Württemberg, Germany) in the 4000–500 cm^−1^ range at a resolution of 4 cm^−1^.

#### 2.2.6. Color Difference Properties

Film color characteristics were measured with a WR18 colorimeter (Shen Zhen Wave Optoelectronics Technology Co., Ltd., Shenzhen, China).

#### 2.2.7. Optical Absorption Analysis

UV–visible absorbance measurements were obtained at 420 nm using a Shimadzu UV–1800 spectrophotometer (Shimadzu Corporation, Kyoto, Japan).

#### 2.2.8. Photothermal Measurement

Film photothermal responses were assessed with an infrared thermal camera (TiS55, Fluke Corporation, Everett, WA, USA).

### 2.3. Synthesis of Dual Metal-Doped CuO Nanoparticles (M-CuO NPs)

A 500 mL solution of 11.35 g (94 mM) copper nitrate was prepared, followed by the addition of 0.25 g (3 mM) AgNO_3_ and 0.38 g (3 mM) Mg(NO_3_) to achieve a Ag:Mg:Cu molar ratio of 3:3:94. The mixture was stirred for 30 min before adding 500 mL of a 25 g PEG solution and stirring for an additional 30 min. Next, 500 mL of a 16 g NaOH solution was added dropwise, and the reaction was maintained at 80 °C for 4 h, followed by 1 h of stirring without heat. The resulting precipitate was centrifuged at 5000 rpm, washed with DI water and ethanol, and the obtained sample was dried at 100 °C overnight. The dried sample was ground using a mortar and pestle and then calcined at 500 °C for 4 h. The obtained sample was referred to as M-CuO NPs [29].

### 2.4. Surface Functionalization of Dual Metal-Doped Copper Oxide (SF-M-CuO)

In detail, 1 g of dual-metal-doped CuO NPs was dispersed in 1 L of DI water and stirred at 37 °C for 1 h. Then, 1 g of GPTMS was added to the mixture, and the resulting solution was stirred for 24 h. Unreacted GPTMS was removed through centrifugation followed by triple washing with DI water. The collected sample was transferred to a glass Petri dish and dried in a hot-air oven at 60 °C. Finally, the obtained sample was referred to as SF-M-CuO.

### 2.5. Preparation of Composite Films

#### 2.5.1. Preparation of PLA and PLA-CCM Composite Films

PLA-CCM composite films were prepared using the solvent-casting method. Initially, 4 g of PLA was slowly added to 100 mL of CHCl_3_ under stirring for 1 h. The mixture was then stirred for an additional 5 h at 37 °C until fully dissolved. Different amounts of CCM (0.25, 0.50, 0.75, 1, 2, 3, and 4 Wt.% based on PLA) were added to 100 mL of CHCl_3_ and dissolved under stirring for 1 h. Subsequently, 4 g of PLA was slowly added to the CCM solution and dissolved for 1 h with vigorous stirring. After complete dissolution, the mixture was stirred for an additional 5 h at 37 °C. Each film-forming solution (approximately 32 g) was then cast onto a glass Petri dish (150 mm × 15 mm) and dried at 37 °C ± 1 °C for 72 h. The dried films were carefully peeled off the plate and conditioned in a humidity chamber at 25 °C and 50% relative humidity (RH) for at least 72 h.

#### 2.5.2. Preparation of PLA-CCM-SF-M-CuO Composite Films

For composite films preparation, the 4% of CCM was dissolved in 100 mL of CHCl_3_, and different concentrations of 1, 2, 3 and 4% SF-M-CuO were added based on PLA content. The process of sonication each mixture for 2 min to ensure uniform dispersion. Furthermore, the 4 g PLA was added to the mixture solution and stirred for 1 h to achieve complete dissolution. The process further stirred the solution at 37 °C for 5 h. The obtained mixture was poured into a glass Petri dish and dried at 37 °C for 72 h. The dried films were peeled off and stored at 25 °C and 50% RH for 72 h.

### 2.6. Color Analysis

The color properties of all composite films using the L^*^, a^*^ and b^*^ color system. The parameter L^*^ indicates lightness, a^*^ represents the red-green coordinate, and b^*^ denotes the yellow-blue coordinate. Then, calculated the total color difference (Δ*E*) using equation (Equation (1)).

(1) $$∆E=\sqrt{{\left(∆L^{*}\right)}^{2}{+(∆a^{*})}^{2}{+(∆b^{*})}^{2}}$$

where *L*_0_^*^, *a*_0_^*^, and *b*_0_^*^ are the color coordinates of the neat PLA film, and *L*_1_^*^, *a*_1_^*^, and *b*_1_^*^ are the corresponding color coordinates of the composite films [41].

### 2.7. Radical Scavenging Activity and Curcumin Release of Composite Films

#### 2.7.1. ABTS Assay of Composite Films

The radical scavenging activity of film samples was evaluated using the ABTS assay, following the protocol described by Eze et al. with minor modifications [42]. Initially, the ABTS cation stock solution was freshly prepared by mixing 5 mL of ABTS solution (7 mM) with 5 mL of potassium persulfate (2.45 mM) and incubating the mixture in the dark for 12 h at 37 °C. The working cation solution was then prepared by diluting the stock solution with 50% ethanol to achieve an absorbance of 0.70 ± 0.02 at a wavelength of 734 nm. Meanwhile, 50 mg of film samples were carefully placed into vials, and 10 mL of the ABTS working solution was added to each sample. The films were incubated for 30 min. ABTS solution without any film sample was used as the control, and parallel samples with only 50% ethanol solution were used as blanks. The ABTS radical scavenging activity (RSA) of the films was calculated using the following equation (Equation (2)):

(2) ABTS RSA (%) = [(A0−A1)/A0]×100

where A0 and A1 are the absorbance values of the control and test samples, respectively.

#### 2.7.2. DPPH Assay of Composite Films

For the DPPH radical scavenging assay, a 1 mM DPPH stock solution was prepared in absolute ethanol. A working DPPH solution (0.1 mM) was then prepared by diluting the stock with 50% ethanol. Film samples (50 mg each) were placed into vials, and 10 mL of the working DPPH solution was added to each. For blanks, corresponding film samples were treated with 10 mL of 50% ethanol solution, and the control consisted of a DPPH solution in 50% ethanol. All samples were incubated in the dark at room temperature for 60 min. After incubation, aliquots were transferred to 96-well microplates, and absorbance was measured at 517 nm [42]. The DPPH RSA was calculated using the following equation (Equation (3)):

(3) DPPH RSA (%) = [(A0−A1)/A0]×100

where A0 and A1 are the absorbance values of the control and test samples, respectively.

#### 2.7.3. Curcumin Release Assay of Composite Films

The curcumin release behavior of the composite films was evaluated using a previously reported method with minor modifications by Roy and Rhim [23]. Each film sample (2 cm × 2 cm) was immersed in 20 mL of DI water in a 55 mL boiling tube and incubated at 37 °C under continuous shaking at 100 rpm. At predetermined time intervals (every 6 h, from 0 to 96 h), 2 mL aliquots of the release medium were withdrawn, and the absorbance was measured at 420 nm using a UV–Vis spectrophotometer. The released curcumin content was quantified using a standard calibration curve and expressed as micrograms of curcumin released per square millimeter of film area (µg/mm^2^).

### 2.8. Cytotoxicity Assay of Composite Films

Mammalian macrophage RAW 264.7 cells were cultured in Dulbecco’s Modified Eagle’s Medium supplemented with 10% fetal bovine serum at 37 °C and 5% CO_2_, seeded at 5 × 10^4^ cells mL^−1^ in 96-well plates, and incubated for 24 h upon reaching approximately 95% confluence. Concurrently, 5 mm diameter disks were punched from each composite film and disinfected by washing with sterile water [43]. The RAW 264.7 cells were then seeded on top of the disks and cultured for 24 h under standard conditions. The untreated film served as the control. Viability was assessed using an EZ-Cytox kit (DoGenBio Co., Ltd., Seoul, Republic of Korea), and absorbance was measured at 450/600 nm.

### 2.9. Photothermal Assay of Composite Films

The infrared images of the PLA, PLA-4%-CCM, and 1–4% of SF-M-CuO incorporated PLA-4%-CCM composite films surfaces were captured under NIR light source (808 nm, 1 W/cm^2^) irradiation with 0.6 mL of DI water in a 24-well plate [44].

### 2.10. Antibacterial Assay of Composite Films

The antibacterial effectiveness of PLA, PLA-4%-CCM, and 1–4% of SF-M-CuO incorporated PLA-4%-CCM composite films was evaluated against the foodborne pathogens *Staphylococcus aureus* (*S. aureus*) and *Escherichia coli* (*E. coli*) using a modified plate count method based on the protocol of Roy and Rhim [23]. Briefly, *S. aureus* and *E. coli* were cultured in trypticase soy broth and nutrient broth, respectively, and incubated at 37 °C for two consecutive subcultures of 24 h. All film samples (3 cm × 3 cm) were placed in flasks containing 10 mL of bacterial suspension (8~9 log CFU/mL) prepared in Muller-Hinton broth. The flasks were incubated at 37 °C with constant agitation at 120 rpm. After 24 h, bacterial counts were determined by plating on trypticase soy agar (for *S. aureus*) and MacConkey agar (for *E. coli*). The plates were incubated at 37 °C for 24 h, after which colony-forming units (CFUs) were enumerated. PLA film is considered a blank for antibacterial activity. The antibacterial activity (R) was calculated using the following equation (Equation (4)):

(4) R (%)=(B−C)/B×100

where B represents the CFU count of the control group, and C represents the CFU count of the treated group after 24 h. In addition, the antibacterial efficacy was further expressed as log10 reduction, which was calculated using the following equation (Equation (5)):

(5) Log Reduction=log10(N0/N)

where N0 is the CFU count of the PLA control film, and N is the CFU count of the treated sample after 24 h of incubation. All experiments were conducted in triplicate to ensure reproducibility.

### 2.11. Statistical Analysis

Data are presented as mean ± standard error of the mean based on three independent experiments. Group comparisons were performed using one-way ANOVA, while paired *t*-tests were applied for two-group analyses. Statistical significance was defined at *p* ≤ 0.05. All analyses were conducted using IBM SPSS Statistics software (Version 26; IBM Corp., Armonk, NY, USA) and GraphPad Prism (Version 8; GraphPad Software Inc., San Diego, CA, USA).

## 3. Results and Discussion

### 3.1. Evaluation of Physicochemical Properties of M-CuO NPs

**Figure 1:**
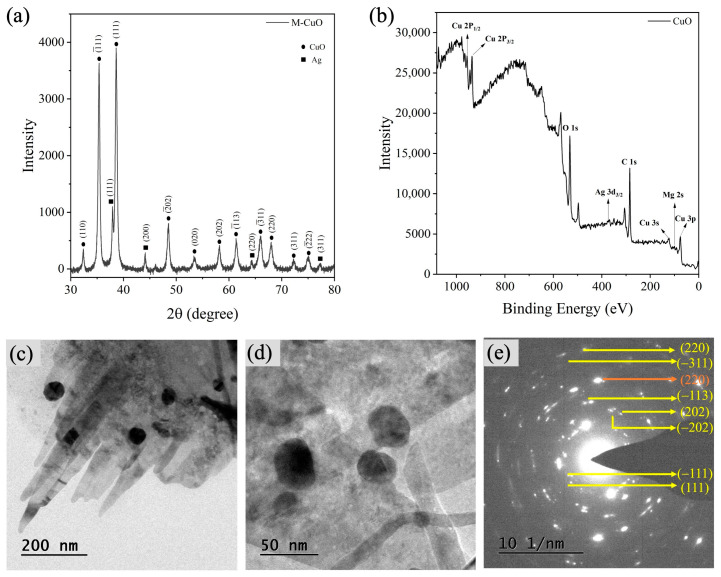
Structural and compositional characterization of synthesized dual-metal-doped copper oxide nanoparticles: (**a**) XRD pattern; (**b**) XPS survey spectrum; (**c**,**d**) TEM images and (**e**) SAED pattern.

**Figure 2:**
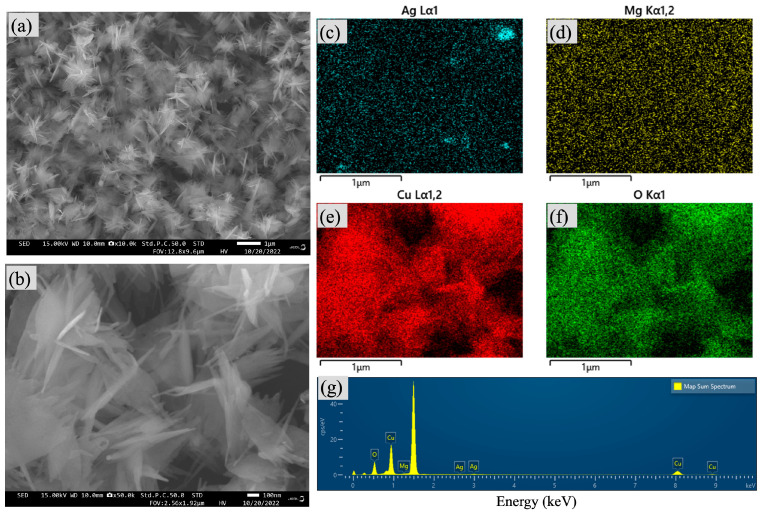
Morphology and elemental mapping analysis of synthesized dual-metal-doped copper oxide nanoparticles: (**a**,**b**) SEM micrographs; (**c**) Ag; (**d**) Mg; (**e**) Cu; (**f**) O elemental mapping images, and (**g**) EDX spectrum.

As shown in Figure 1a, the XRD analysis confirmed that M-CuO NPs formed a monoclinic CuO crystal structure, as indicated by characteristic diffraction peaks matching JCPDS No. 80–1916 (the circle symbol). The XRD pattern also revealed face-centered cubic Ag peaks (JCPDS No. 04–0783), confirming the formation of metallic Ag NPs during synthesis (square symbol). No distinct Mg-containing crystalline phases were detected, suggesting possible incorporation of Mg into the CuO structure at concentrations below the XRD detection limit. It has an average crystallite size of 46.66 nm [29]. The XPS wide-scan spectrum identified Cu, O, Ag, C, and Mg elements, confirming the presence of Ag and Mg in the synthesized CuO NPs (Figure 1b). As shown in Figure 1c,d, TEM analysis showed that Ag NPs decorated the CuO surface, whereas Mg species were associated with the CuO phase, which promoted anisotropic growth and resulted in a nanorod-like morphology. In Figure 1e, the SAED pattern displayed polycrystalline ring structures and confirmed the presence of metallic Ag through the (220) diffraction plane [29]. The sample exhibited an average length of 702.67 nm and a width of 58.71 nm. As depicted in Figure 2a,b, SEM images revealed that the M-CuO NPs formed agglomerated flake-like structures, while elemental mapping confirmed the uniform distribution of Ag, Mg, Cu, and O elements (Figure 2c–f). EDX analysis further verified the elemental composition, showing characteristic peaks for O, Cu, Mg, and Ag (Figure 2g). In Table S1, the quantitative analysis indicated weight percentages of 1.90% Ag, 2.73% Mg, 72.44% Cu, and 22.94% O, confirming the presence of Ag decoration and Mg incorporation in the CuO NPs [29].

### 3.2. Functional Analysis of M-CuO NPs, GPTMS, SF-M-CuO and CCM

**Figure 3:**
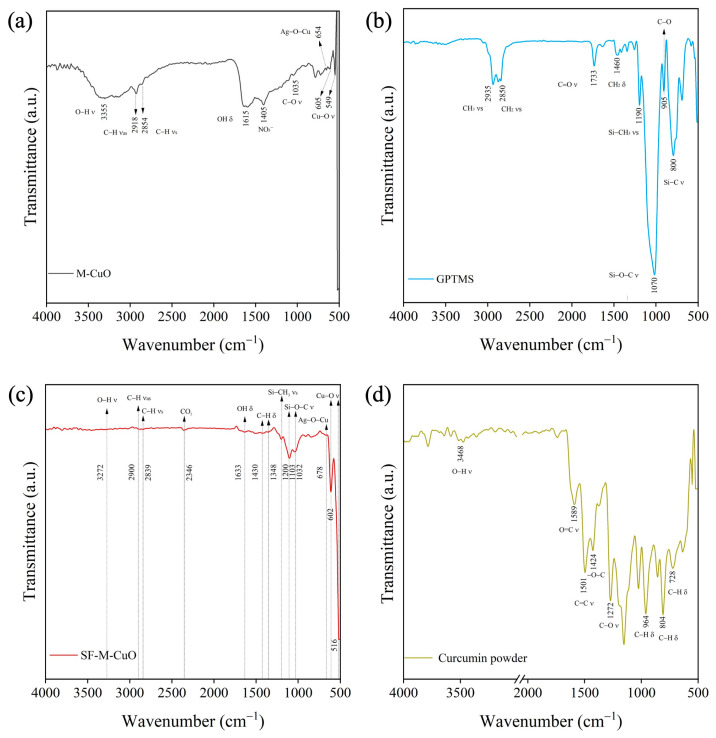
FTIR spectra of (**a**) synthesized dual-metal-doped copper oxide nanoparticles; (**b**) GPTMS; (**c**) surface-functionalized dual-metal-doped copper oxide, and (**d**) curcumin powder.

As shown in Figure 3a, the FTIR spectrum of calcined M-CuO NPs. The spectrum displays a broad O–H stretching band at 3355 cm^−1^, confirming surface hydroxyl groups. Peaks at 2918 and 2854 cm^−1^ indicate C–H stretching vibrations, while the band at 1615 cm^−1^ corresponds to O–H bending. The peak at 1405 cm^−1^ represents asymmetric nitrate (NO_3_^−^) stretching, and the band at ~1035 cm^−1^ indicates C–O vibrations from PEG [27,29]. The peak at 654 cm^−1^ corresponds to Ag–O–Cu bonding, and bands at 605 and 549 cm^−1^ confirm Cu–O stretching, verifying the formation of M-CuO NPs [29]. Figure 3b presents the FTIR spectrum of GPTMS. The spectrum shows characteristic bands at 2935 and 2850 cm^−1^ for C–H stretching, 1733 cm^−1^ for C=O stretching, 1460 cm^−1^ for CH_2_ bending, 1190 cm^−1^ for Si–CH_3_ stretching, 1070 cm^−1^ for Si–O–C stretching, 905 cm^−1^ for epoxide ring deformation, and 800 cm^−1^ for Si–C stretching [45,46,47]. Figure 3c displays the FTIR spectrum of SF-M-CuO. The spectrum shows O–H stretching at 3272 cm^−1^, indicating hydroxyl and silanol groups. Peaks at 2900 and 2839 cm^−1^ represent C–H stretching, while the band at 2346 cm^−1^ corresponds to adsorbed CO_2_. The peak at 1633 cm^−1^ suggests carbonyl groups, and bands at 1430 and 1348 cm^−1^ indicate C–H bending. Peaks at 1200, 1103, and 1032 cm^−1^ confirm Si–O–CH_3_ and Si–O–C bonding from GPTMS. The Ag–O–Cu vibration appears at 678 cm^−1^, and Cu–O stretching bands at 602 and 516 cm^−1^ confirm the CuO material. Figure 3d showed the FTIR spectrum of the CCM powder sample. The broad peak at 3468 cm^−1^ corresponds to phenolic O–H stretching vibrations, while the band at 1589 cm^−1^ is assigned to C=O stretching of the conjugated diketone structure. The peak at 1501 cm^−1^ arises from aromatic C=C stretching, and the band at 1424 cm^−1^ indicates C–O stretching of phenolic groups. The absorption at 1272 cm^−1^ corresponds to C–O stretching of ether linkages, whereas peaks at 964, 804, and 728 cm^−1^ are attributed to C–H out-of-plane bending of alkene and aromatic groups. These results agree well with the previous literature reported [23].

### 3.3. Visual Appearance and Color Difference Analysis of Composite Films

**Figure 4:**
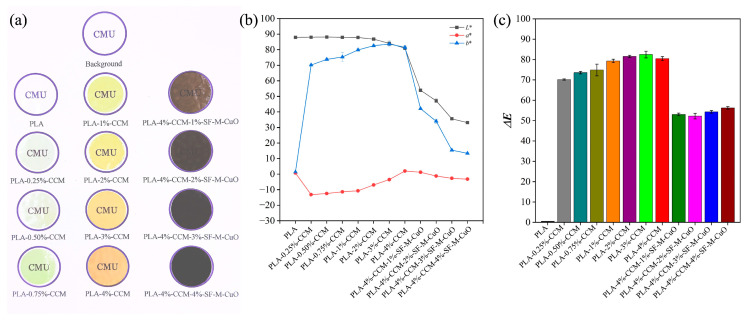
Optical properties of composite films: (**a**) photographic images, (**b**) L^*^, a^*^, and b^*^ color parameters, and (**c**) total color difference (Δ*E*).

The visual appearance of the composite films (⌀ 2.5 cm) is shown in Figure 4a. The PLA film exhibits high transparency. Increasing CCM concentration enhances film yellowness and leads to the selection of PLA-4%-CCM as the base matrix due to its uniform color. Incorporation of 1–2%-SF-M-CuO produces acceptable darkening, whereas 3–4%-SF-M-CuO causes excessive opacity. The L^*^, a^*^, and b^*^ color coordinates of the films are shown in Figure 4b. PLA exhibits a high L^*^ value with near-zero a^*^ and b^*^ values, confirming the transparency of the PLA film. Increasing CCM concentration in the PLA film decreases L^*^ and increases b^*^, indicating enhanced yellowness due to CCM. The addition of SF-M-CuO to PLA-4%-CCM further decreases L^*^ and slightly reduces b^*^, suggesting partial masking of the CCM color. Kim et al. reported that incorporating rod-like CuO NPs (50–130 nm) at 0.5–1.5 Wt.% into PLA films caused progressive darkening and a significant reduction in L^*^ values as the CuO content increased [48]. Figure 4c reveals the total color difference (Δ*E*) of the composite films. The Δ*E* values increase from 0.00 (PLA) to 82.48 (3% CCM), indicating substantial color variation. A slight decrease to 80.47 at 4% CCM suggests a saturation effect, where additional CCM has a diminished influence on color. Roy and Rhim reported that PLA composite films containing CCM (0.25–1.50 Wt.%) exhibited decreased L^*^ and a^*^ values and increased b^*^ values with increasing CCM concentration, resulting in a Δ*E*, which was mainly attributed to enhanced film yellowness induced by CCM incorporation [23]. In contrast, films containing SF-M-CuO exhibit lower Δ*E* values (52.22–56.22) than PLA and PLA-CCM films, indicating that CCM primarily governs the visual properties of the films. The less pronounced effect of SF-M-CuO is attributed to its uniform dispersion within the polymer matrix, which reduces overall color variation.

### 3.4. FTIR Analysis of Composite Films

**Figure 5:**
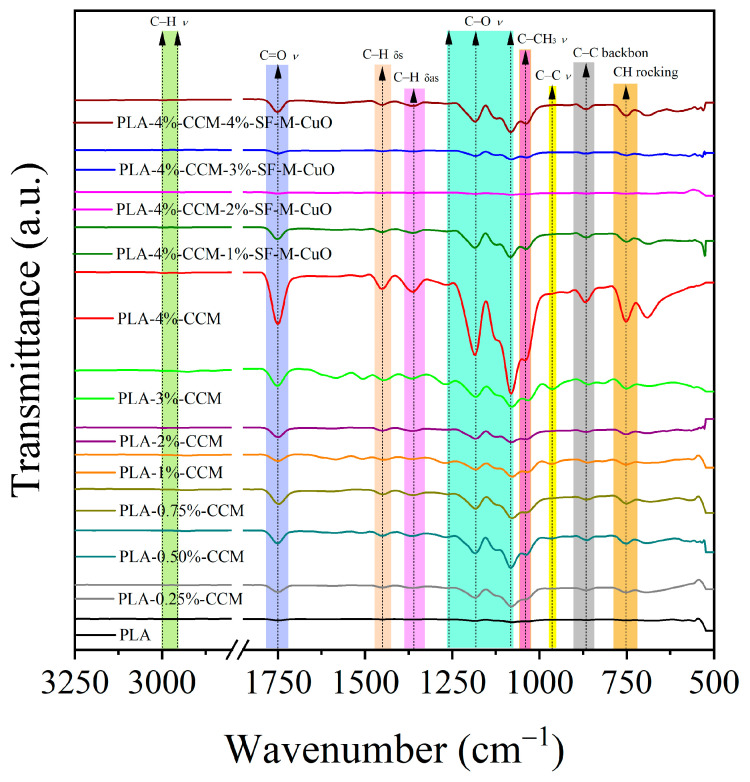
FTIR spectra of composite films.

In Figure 5, the FTIR spectrum of PLA showed characteristic bands at 3000 and 2950 cm^−1^ (C–H stretch), 1750 cm^−1^ (C=O stretch), 1452 and 1365 cm^−1^ (C–H deformation), and 1266–1080 cm^−1^ (C–O stretch). It also showed peaks at 1042 cm^−1^ (C–CH_3_ stretch), 955 cm^−1^ (C–C stretch), and 869–754 cm^−1^ (C–C/CH rock) [31,49]. The addition of CCM (0.25–4%) to PLA showed no new peaks, and no peak shifts were observed in the FTIR spectrum. However, the intensities of the existing peaks increased progressively with increasing CCM content (1–4%), reaching their maximum at 4% as a result of enhanced physical interactions within the PLA matrix. Roy and Rhim reported that PLA-CCM composite films exhibited no new FTIR peaks and showed only slight changes in peak intensities, indicating the absence of new chemical bond formation and confirming physical interactions between PLA and CCM [23]. Furthermore, the PLA-4%-CCM composite was further modified with SF-M-CuO (1–4%). No significant spectral changes were observed at 1% and 4% SF-CuO, indicating weaker intermolecular interactions at these concentrations (Figure 5). In contrast, the 2% and 3% SF-M-CuO composites showed reduced intensity at 1750, 1266, 1180, and 1080 cm^−1^, revealing stronger physical interactions. These results suggest that 2–3% SF-M-CuO provides optimal dispersion and interaction, while higher loading (4%) leads to SF-M-CuO aggregation. Kim et al. reported that PLA bionanocomposite films containing different contents of ZnO NPs showed no significant shifts in FTIR peak positions, indicating the absence of strong chemical interfacial interactions between the PLA matrix and ZnO NPs [31].

### 3.5. XRD Analysis of Composite Films

**Figure 6:**
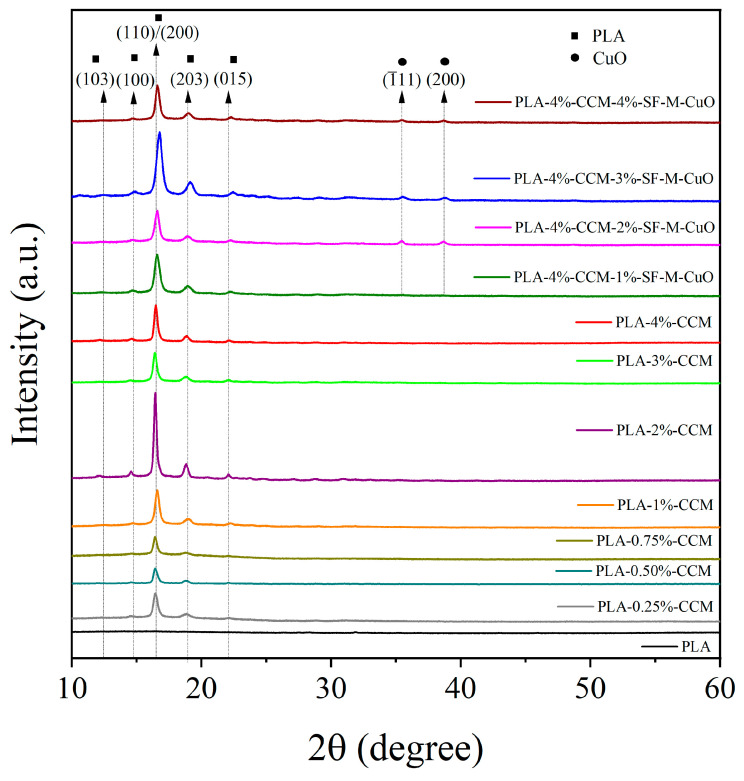
XRD patterns of composite films.

The XRD patterns of all the composite films are presented in Figure 6. The XRD pattern of the PLA film exhibits a broad halo between 12.0° and 22.5°, indicating the amorphous nature of PLA. This lack of crystallinity is attributed to the rapid solvent evaporation during the solution casting process, which inhibits the orderly arrangement of polymer chains [50,51]. As shown in Figure S1, the XRD analysis of CCM powder revealed intense diffraction peaks at 2θ = 17.33°, 24.59°, and 25.62°, with other significant peaks observed at 12.18°, 14.51°, 21.13°, 23.32°, 26.10°, and 27.43°. All peaks correspond to the characteristic lattice planes of crystalline CCM [52]. The incorporation of CCM into the PLA matrix at concentrations of 0.25–4% promoted crystallinity of PLA, as evidenced by the appearance of characteristic XRD peaks at 2θ = 12.5°, 14.8°, 16.8°, 19.1°, and 22.3°. These peaks correspond to the (103), (100), (110)/(200), (203), and (015) planes of the α-crystalline form of PLA, confirming that CCM acts as a nucleating agent [23]. Yang et al. reported that an aromatic sulfonate nucleating agent increased the crystallinity of PLA composites and induced the appearance of a new (203) crystal peak in the XRD pattern [53]. Furthermore, the incorporation of 1–4% SF-M-CuO into the PLA-4%-CCM matrix enhanced the intensity of characteristic PLA crystalline planes (103), (100), (110)/(200), (203), and (015), indicating improved crystallinity. This effect was most pronounced at 3% SF-M-CuO, beyond which the peak intensity at 4% decreased, suggesting reduced crystallinity. Additional peaks at 35.47° and 38.69°, corresponding to the (-111) and (200) planes of CuO, confirmed the successful integration of SF-M-CuO and its role in promoting crystalline order (Figure 6).

These findings indicate that 3% of SF-M-CuO acts as an effective nucleating agent, while the decrease in crystallinity at 4% loading is likely due to SF-M-CuO aggregation. which disrupts the intermolecular interactions between PLA chains, reducing their regularity and overall crystallinity. Kim et al. reported that low ZnO NPs contents (0.5–1%) increased PLA peak intensities and enhanced crystallinity by acting as nucleating agents. Higher ZnO NPs loadings (>3–10%) reduced PLA crystallinity because NPs agglomeration disrupted PLA chain regularity [31], which agrees with the trends observed in the present study. At the same time, the crystallinity index (CrI) of the synthesized composite films was determined using equation (Equation (6)) [42].

(6) $$Crystallinity\;Index\;(\%)=[(I_{Cr}/(I_{Cr}+I_{non-Cr})]\times 100$$

where I*_Cr_* and I*_non−Cr_* represent the integrated intensities of the crystalline and non-crystalline (amorphous) phases, respectively.

**Figure 7:**
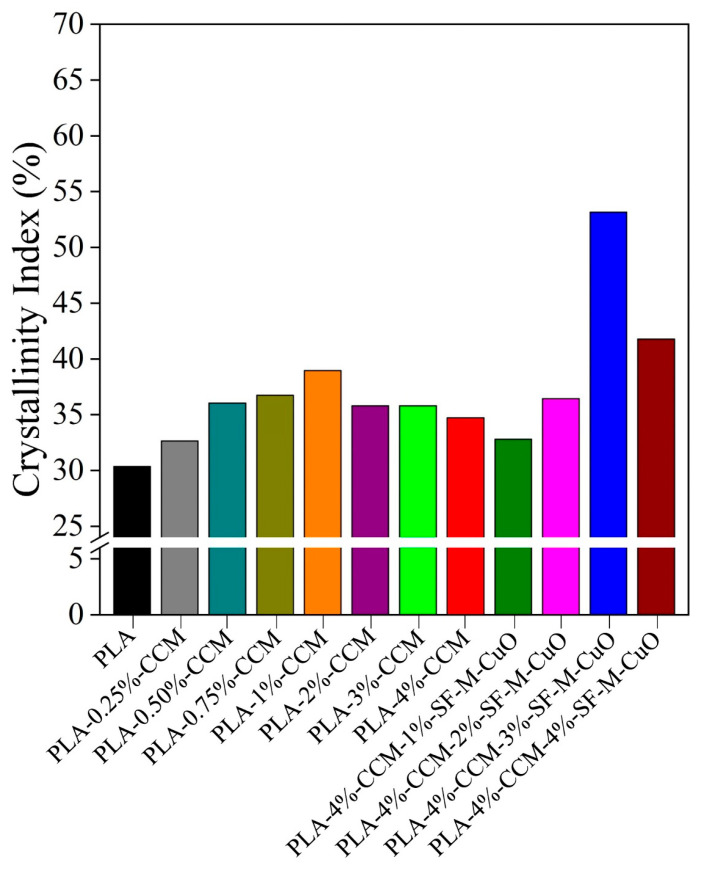
Crystallinity index (CrI) of composite films.

As shown in Figure 7, the PLA film exhibits a low CrI at 30.35%, which is characteristic of its amorphous nature, as confirmed by a broad XRD peak. The incorporation of CCM (0.25–4%) increases the CrI of PLA to 32.65–38.94%, indicating that CCM promotes PLA CrI. Roy and Rhim reported that the CrI of the PLA film increased to 29.9% with the addition of 1.5 Wt.% CCM, which acts as a nucleating agent in PLA [23]. Mondal et al. found that a 2% CCM additive raised the CrI of PLA films from 37% to 51%, enhancing PLA crystallinity [54]. The addition of SF-M-CuO (1–4%) to the PLA-4%-CCM matrix further modifies CrI, with the maximum CrI (53.16%) observed at 3% SF-M-CuO. However, 1% SF-M-CuO slightly reduces CrI compared to PLA-4%-CCM, suggesting concentration-dependent nucleation and dispersion effects. The SF-M-CuO enhance polymer crystallization by acting as nucleating agents, while excessive loading or poor dispersion hinders chain packing and reduces crystallinity depending on the NPs content, dispersion, and interfacial interactions [31].

### 3.6. Surface Morphology, Elemental Mapping, and EDX Profile Analysis of Composite Films

**Figure 8:**
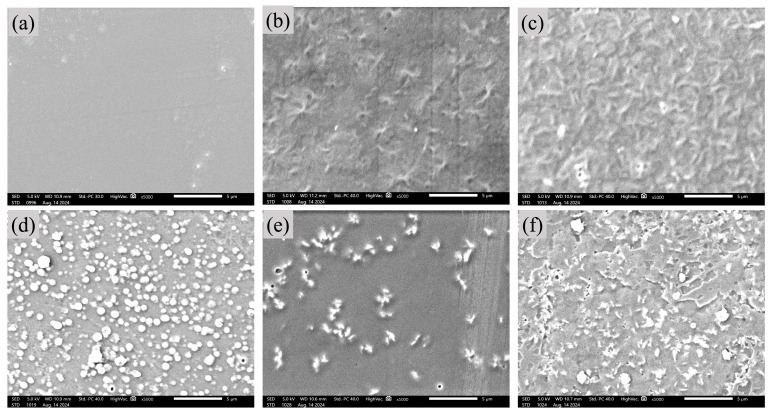
SEM analysis of composite films: (**a**) PLA; (**b**) PLA-4%-CCM; (**c**) PLA-4%-CCM-1%-SF-M-CuO; (**d**) PLA-4%-CCM-2%-SF-M-CuO; (**e**) PLA-4%-CCM-3%-SF-M-CuO, and (**f**) PLA-4%-CCM-4%-SF-M-CuO.

**Figure 9:**
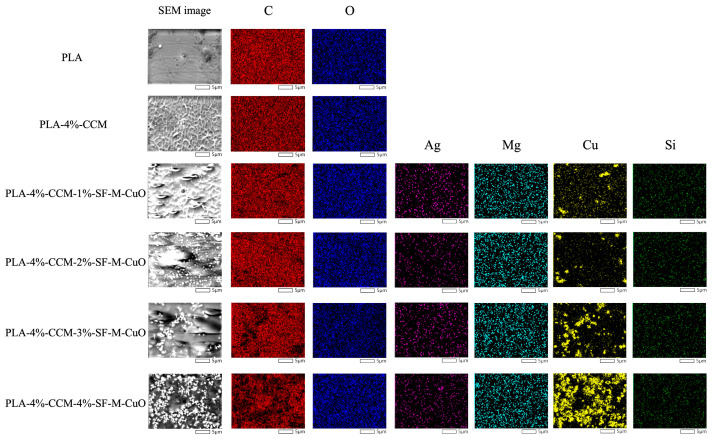
SEM micrographs showing the surface morphology and corresponding elemental mapping profiles of the composite films.

As shown in Figure 8, SEM analysis reveals the morphological evolution of the composite films. The PLA film exhibits a characteristically smooth and homogeneous surface (Figure 8a). The incorporation of 4% CCM introduces distinct surface irregularities, indicating its successful integration into the polymer matrix (Figure 8b). With the addition of SF-M-CuO, a concentration-dependent dispersion behavior is observed: films with 1% and 2% SF-M-CuO show partial dispersion (Figure 8c,d), while 3% SF-M-CuO results in uniform dispersion with minimal aggregation, suggesting optimal integration and effective nucleation (Figure 8e). Conversely, the 4%-SF-M-CuO composite exhibits significant agglomeration, compromising film homogeneity and interfacial adhesion (Figure 8f). These results demonstrate that 3% SF-M-CuO represents the optimal concentration for enhancing morphological properties. As shown in Figure 9, elemental mapping confirms the successful incorporation and uniform dispersion of SF-M-CuO within the PLA-4%-CCM matrix. The uniform carbon (C) distribution indicates a continuous and intact polymer matrix. Oxygen (O) is ubiquitous, originating from the polymer chains and the SF-M-CuO. The distinct and evenly distributed copper (Cu) signal verifies effective nanoparticle dispersion, while the presence of silicon (Si) confirms the successful silanization functionalization via GPTMS. Trace amounts of magnesium (Mg) and silver (Ag) are also detected, resulting from the dual-metal doping process of the CuO matrix. Collectively, the elemental mapping analysis demonstrates that the functionalized nanomaterials are well-dispersed without disrupting the integrity of the polymer composite. As shown in Table 1, the EDX analysis of the composite films revealed that PLA and PLA-4%-CCM matrices were primarily composed of C (81.83–82.82 Wt.%) and O (17.18–18.17 Wt.%). Incorporating SF-M-CuO significantly altered the elemental composition, with Cu content increasing from 1.11 Wt.% at 1% SF-M-CuO to 20.02 Wt.% at 4% SF-M-CuO. The C and O content slightly decreased, whereas it remained stable at 1% loading. Trace elements from functionalization and doping were detected. The Ag signal, indicative of dual-metal doping, was present at 1.47 Wt.% at 1% SF-M-CuO, decreasing to 0.06 Wt.% at 3% and rising to 2.15 Wt.% at 4% SF-M-CuO. The Mg signal was detected only in the 4% SF-M-CuO sample (0.04 Wt.%). No Si signal was detected, suggesting limitations in detecting the silane layer (Figure S2). These trends suggest that 3% SF-M-CuO promotes uniform dispersion and strong interfacial interactions in the PLA-CCM matrix. However, the significant increase in Ag and Mg signals at the 4% SF-M-CuO concentration, together with high Cu levels, indicates nanomaterial aggregation, which is consistent with the SEM observations. Overall, the EDX results confirm the successful incorporation of SF-M-CuO into the composite films (Figure S2).

### 3.7. Biofunctional Properties of Composite Films

#### 3.7.1. Antioxidant Activity of Composite Films

**Figure 10:**
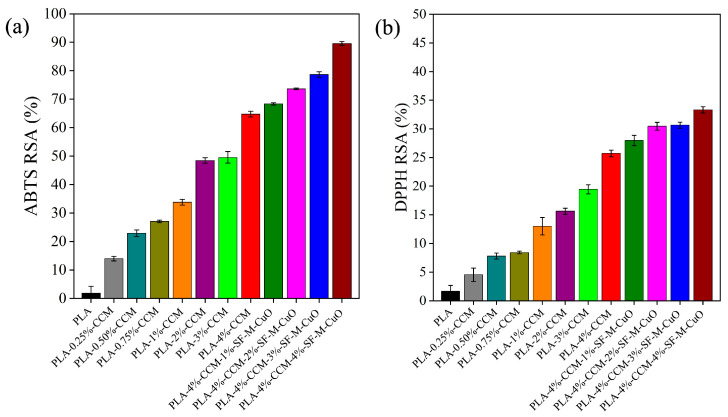
Radical scavenging activity of composite films: (**a**) ABTS assay and (**b**) DPPH assay.

The antioxidant activity of all the composite films was assessed using the ABTS^•+^ and DPPH^•^ assays, as shown in Figure 10a,b. The ABTS and DPPH^•^ activities of the PLA film were 1.86% and 1.64%, respectively. Incorporating CCM into PLA increased ABTS activity from 13.99% to 68.36% and DPPH activity from 4.53% to 25.71% with increasing CCM content (0.25–4%). Adding (1–4%) of SF-M-CuO to PLA-4%-CCM film further enhanced ABTS (64.77–89.56%) and DPPH (27.97–33.33%) activities. The antioxidant activity of the PLA-CCM composite films arises from the presence of CCM, which scavenges ABTS^•+^ and DPPH^•^ radicals through hydrogen atom transfer and single-electron transfer mechanisms due to its phenolic hydroxyl groups and conjugated structure [23,54]. Roy and Rhim reported that neat PLA films exhibited low DPPH and ABTS scavenging activities (1.8% and 3.1%, respectively), which significantly increased to 76.6% and 94.7% upon the incorporation of 1.5 Wt.% curcumin [23]. Previously, the PLA and PLA-CCM composite film increased DPPH radical scavenging activity from 16.8% to 83%. CCM enhances antioxidant activity by donating hydrogen atoms from its phenolic groups, which effectively neutralize free radicals [54]. The addition of SF-M-CuO enhances antioxidant performance through synergistic surface-mediated electron transfer within the polymer matrix, thereby promoting sustained antioxidant activity. The PLA-CCM–SF-M-CuO composite films protect oxidation-sensitive foods by preventing oxidative degradation and extending shelf life.

#### 3.7.2. Curcumin Release Profile of Composite Films

**Figure 11:**
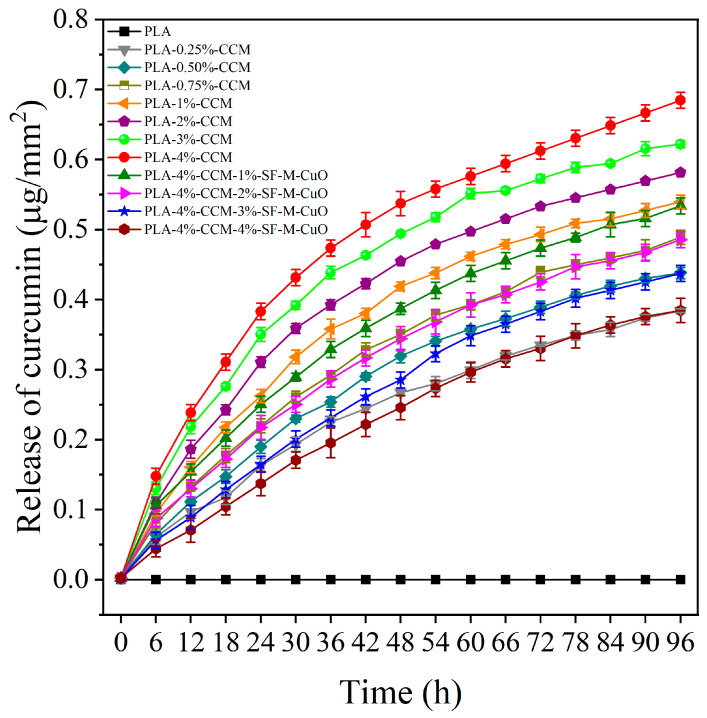
Time-dependent curcumin release profile of composite films.

The curcumin release profile of the composite films is shown in Figure 11. The PLA film did not exhibit any detectable curcumin release over the 0–96 h period. As expected, PLA composite films containing 0.25–4% CCM showed a gradual and time-dependent increase in curcumin release. Notably, the PLA-4%-CCM films exhibited an initial release of approximately 0.14 µg/mm^2^ within the first 6 h, reaching a maximum of 0.68 µg/mm^2^ after 96 h. Previously, Roy and Rhim reported that PLA composite films containing 0.25% and 1.50% curcumin showed low and maximum release behavior, respectively [23]. The curcumin release profiles observed for the PLA-CCM composite films in the present study are consistent with these findings. In contrast, PLA-4%-CCM composite films incorporating SF-M-CuO exhibited a slower curcumin release. The PLA-4%-CCM films containing 1%, 2%, 3%, and 4% SF-M-CuO showed release values of 0.53, 0.48, 0.43, and 0.39 µg/mm^2^ at 96 h, respectively. Accordingly, the release profiles of PLA-4%-CCM films with 1%, 2%, and 3% SF-M-CuO were lower than those of PLA composite films containing 1%, 0.75%, and 0.50% CCM, respectively. Notably, the curcumin release behavior of the PLA-4%-CCM-4%-SF-M-CuO film closely resembled that of the PLA-0.25%-CCM composite film. This reduction in curcumin release is attributed to enhanced interfacial interactions between SF-M-CuO and the PLA-CCM polymer matrix, which reduces polymer chain mobility and promotes tighter packing of curcumin within the composite film. As the SF-M-CuO content increases, the diffusion rate of curcumin decreases. Therefore, the incorporation of SF-M-CuO into the PLA-CCM matrix effectively retards the curcumin release profile (Figure 11).

#### 3.7.3. Biocompatibility of Composite Films

**Figure 12:**
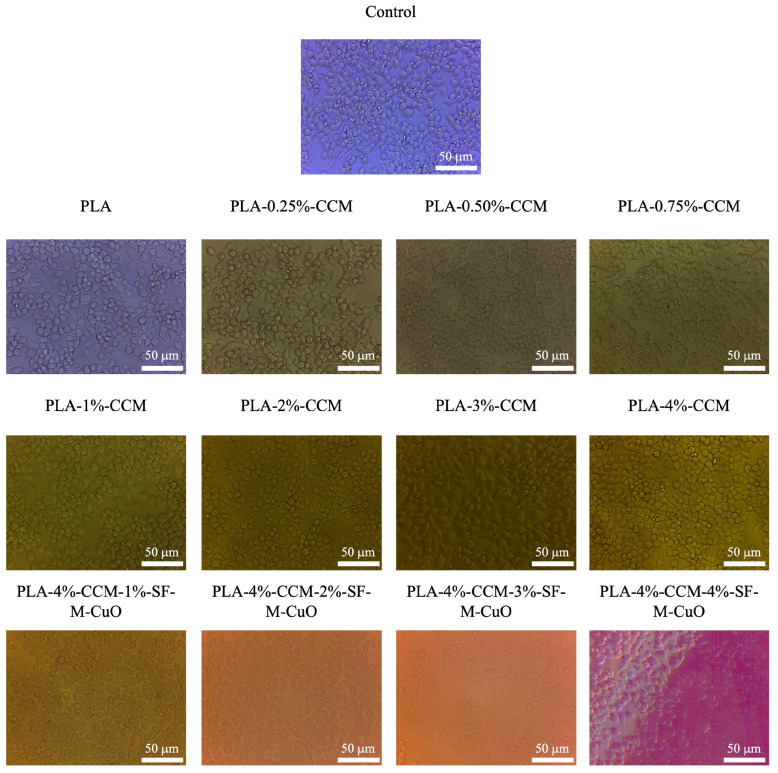
Biocompatibility evaluation of composite films using RAW 264.7 macrophage cells: Representative microscopic images after 24 h incubation.

**Figure 13:**
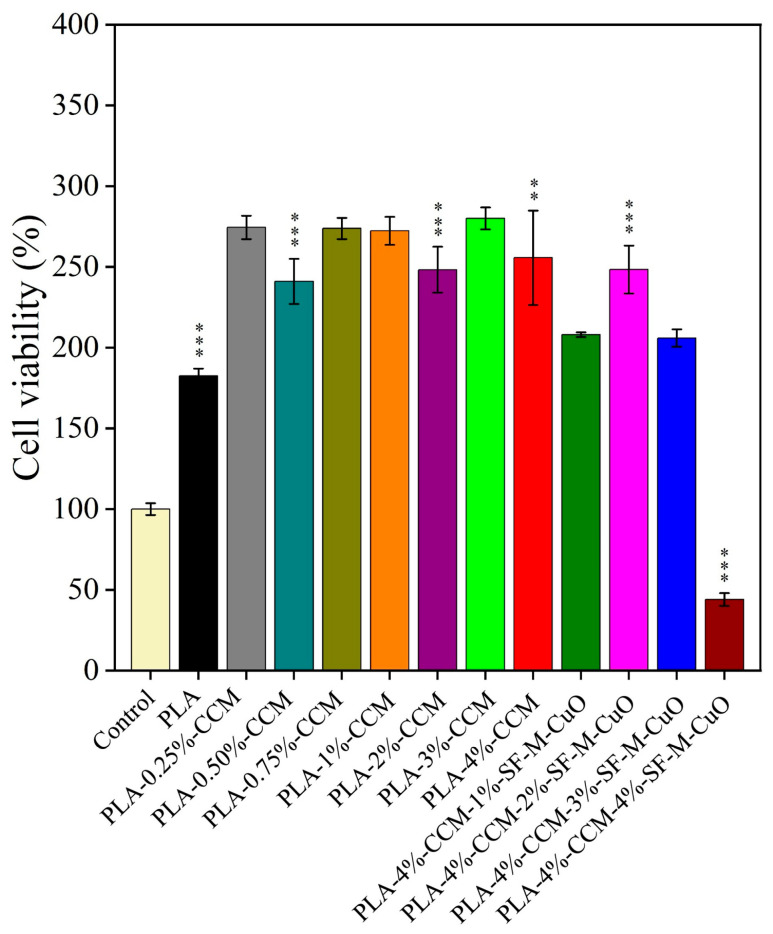
Biocompatibility evaluation of composite films using RAW 264.7 macrophage cells: Quantitative cell viability analysis of composite films. Statistical significance was evaluated using an independent samples *t*-test (** *p* < 0.01, *** *p* < 0.001) compared to the control.

Biocompatibility assessment is vital for appraising the biosafety of CuO and metal-doped CuO in food packaging and biomedical fields [55,56,57,58,59]. As shown in Figure 12, the composite films were evaluated for cell viability using RAW 264.7 macrophage cells, and microscopic images were recorded after 24 h of incubation. In Figure 13, the control group (without film) showed baseline cell viability of 100%. Neat PLA increased cell viability to 182.62%, confirming its non-cytotoxic nature. Incorporation of 0.25–4% CCM into PLA further enhanced cell viability to 241.02–255.66%, indicating that CCM promotes macrophage cell proliferation. In contrast, PLA-4%-CCM films containing SF-M-CuO exhibited concentration-dependent effects. Films with 1–3% SF-M-CuO maintained high cell viability (205.94–248.32%), suggesting a synergistic interaction between CCM and SF-M-CuO at moderate loadings. However, the film containing 4% SF-M-CuO showed a marked reduction in cell viability (44.08%), which is attributed to excessive nanofiller loading and reduced curcumin availability, leading to moderate cytotoxic effects. In general, materials that exhibit cell viability above 90% are considered non-cytotoxic and suitable for cell growth applications. Jiao et al. reported that non-cytotoxic 10 and 100 nm Ag NPs at 1–2 mg/L increased human hepatoma cell viability to 125–150% after 48–72 h compared to the control, indicating enhanced cell proliferation at low doses, whereas Ag^+^ did not show cytotoxic effect [60]. Patel et al. reported that MgO NPs (30 nm) showed no significant cytotoxicity up to 350 µg/mL in the human intestinal cell and up to 250 µg/mL in cervical cancer cells [61]. In addition, Ag- and Mg-doped CuO nanocomposites (3:3:94) maintained RAW 264.7 cell viability above 94% at 0.5 µg/mL due to the synergistic ion-release effects of Cu^2+^, Ag^+^, and Mg^2+^ [29]. These findings are consistent with and support the results of the present study. Overall, PLA-4%-CCM composite films containing up to 3% SF-M-CuO demonstrated good biocompatibility, highlighting their potential for food-packaging and biomedical applications.

#### 3.7.4. Photothermal Analysis of Composite Films

**Figure 14:**
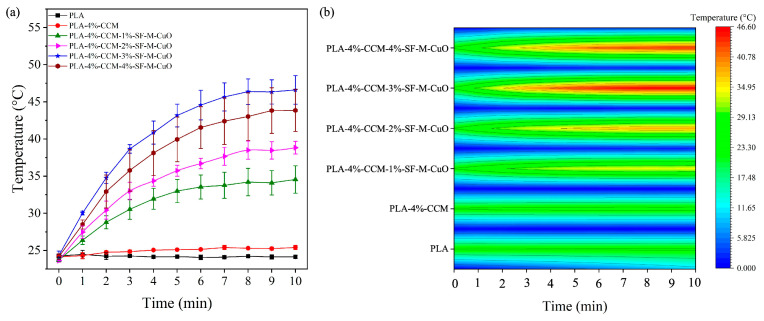
Photothermal performance of composite films under NIR irradiation: (**a**) Time-dependent temperature profiles of composite films exposed to NIR laser irradiation (808 nm, 1 W/cm^−2^) with 0.6 mL of DI water; (**b**) Heat-map analysis showing the corresponding temperature distribution of the composite films over a 0–10 min irradiation period.

Figure 14a presents the photothermal analysis of PLA, PLA-4%-CCM, and 1–4% of SF-M-CuO incorporated PLA-4%-CCM composite films. As shown in Figure 14b, the heat mapping analysis of PLA film exhibited a minimal temperature change from 24.26 to 24.13 °C under NIR irradiation for 10 min, indicating low photothermal activity. The PLA-4%-CCM film showed a slight temperature increase from 24.15 to 25.40 °C, suggesting that CCM marginally enhances the thermal response of PLA. Incorporation of SF-M-CuO at 1, 2, 3, and 4% led to progressively higher temperature rises of 23.72–34.55 °C, 23.63–38.83 °C, 24.33–46.66 °C, and 24.17–43.85 °C, respectively, within 10 min. This demonstrates that SF-M-CuO improves PLA film photothermal efficiency, due to the SF-M-CuO strong NIR absorption. Osman et al. reported that under irradiation with an 808 nm laser at a power density of 1 W/cm^2^ for 10 min, the temperatures of PLA–dexamethasone sodium phosphate fibers containing copper nanoparticles (PLA-DexP@CuNPs) and PLA-DexP@CuNPs-reduced graphene oxide (rGO) fibers increased from 43.6 °C to 52.4 °C, respectively, whereas the PLLA/DexP fibers alone reached only 33.6 °C. These results confirm that the incorporation of rGO and CuNPs significantly enhances light absorption and photothermal performance [62]. This finding supports the current investigation. Notably, 3% of SF-M-CuO provided optimal interfacial interaction and acted as effective nucleating agents, enhancing PLA crystallinity. In contrast, 4% of SF-M-CuO caused aggregation, reducing dispersion, disrupting intermolecular PLA interactions, and slightly decreasing crystallinity. Overall, SF-M-CuO facilitates efficient photothermal conversion, rendering these films suitable for NIR-induced heat generation applications. A mild hyperthermia of 40–42 °C is safe for cells and can promote biological responses useful in regenerative medicine and cancer treatment at 43–50 °C [63].

#### 3.7.5. Antibacterial Activity of Composite Films

The antibacterial performance of the PLA, PLA-4%-CCM, and 1–4% of SF-M-CuO incorporated PLA-4%-CCM composite films was evaluated using the percentage reduction (R%) of bacterial colonies, as shown in Table 2. After 24 h of incubation, the PLA film showed no reduction effect against *S. aureus* and *E. coli*, which is consistent with previous reports in the literature [64,65,66,67]. When the incorporation of CCM further enhanced antibacterial performance, with bacterial growth reductions of 98.50% against *S. aureus* and 96.45% against *E. coli*, corresponding to log10 reductions of 1.83 and 1.45, respectively. Mondal et al. found that the addition of CCM to the PLA resulted in excellent antibacterial activity with colony inhibition rates of 87% for *E. coli* and 77% for *S. aureus*. CCM inhibits microbial growth by binding to the FtsZ protein, preventing Z-ring formation and cell division. CCM also disrupts the cell membrane and damages the peptidoglycan layer, which further suppresses bacterial viability [54]. The incorporation of SF-M-CuO significantly enhanced the antibacterial performance of the composite films. PLA-4%-CCM-1%-SF-M-CuO achieved bacterial reductions greater than 99.99%, corresponding to log10 reductions of 6.08 and 3.95 against *S. aureus* and *E. coli*, respectively. Further increasing the SF-M-CuO content to 2% resulted in log10 reductions of 8.22 and 5.55 against *S. aureus* and *E. coli*, respectively. For composite films containing 3% and 4% SF-M-CuO, bacterial counts were below the limit of detection (LOD), indicating extremely strong antibacterial activity. The antibacterial activity increased with SF-M-CuO content, confirming the nanomaterial’s role in disrupting bacterial viability. Superior activity against *S. aureus* compared to *E. coli* is attributed to structural differences in their cell walls, where the thick peptidoglycan layer of Gram-positive bacteria favors more effective interaction with Ag and Mg present in the CuO. These results indicate that 3%-SF-M-CuO provides an optimal balance between dispersion and antibacterial efficiency. The enhanced activity likely arises from the synergistic effect of SF-M-CuO with CCM in the PLA matrix, involving disruption of bacterial membranes and generation of reactive oxygen species. Kim et al. reported that PLA composite films containing 0.5–2% CuO NPs exhibited greater bacterial growth inhibition than PLA film, with higher CuO NPs content producing stronger antibacterial effects against *L. monocytogenes* and *E. coli* O157:H7. In addition, lavender essential oil (LEO) at concentrations of 0.5–2% showed concentration-dependent antibacterial activity. The combined presence of CuO NPs and bioactive compound of LEO further enhanced bacterial growth inhibition. The antibacterial activity was more pronounced against *E. coli*, followed by *L. monocytogenes* [48]. Akshaykranth et al. reported that PLA film showed no antibacterial activity, and PLA-CCM film revealed low antibacterial activity. PLA composite films containing equal ratios of CCM and ZnO NPs (0.3–0.9 Wt.%) exhibited dose-dependent antibacterial activity, with higher CCM and ZnO contents producing greater bacterial inhibition of *E. coli* and *S. aureus* [68]. Overall, these findings demonstrate that SF-M-CuO impart significant antibacterial properties to the PLA-CCM matrix, highlighting the potential of these composite films for food packaging and biomedical applications.

## 4. Conclusions

In this study, dual-metal-doped CuO NPs were synthesized using the coprecipitation method and subsequently surface-functionalized with the GPTMS compatibilizer. Furthermore, CCM and SF-CuO were successfully incorporated into the PLA polymer matrix through the solution casting method. Functional, structural, and antioxidant curcumin-release results confirmed that 4% CCM is the optimum concentration for PLA-based composite films. The addition of CCM and SF-M-CuO enhanced PLA crystallinity, with both acting as effective nucleating agents to increase the interfacial interaction and CrI. The ABTS and DPPH results revealed dose-dependent antioxidant activity in the composite films, driven by the synergistic effects of CCM and SF-M-CuO. In addition, the incorporation of SF-M-CuO into the PLA-CCM composite films enabled controlled curcumin release. Demonstrating multiple functionalities, the composites enhanced biocompatibility by promoting proliferation of RAW 264.7 macrophages, exhibited efficient photothermal heating (reaching 46.66 °C within 10 min), and achieved excellent antibacterial activity against both *S. aureus* and *E. coli*, reducing bacterial counts to below the LOD at 3% SF-M-CuO content. Therefore, the PLA-4% CCM-3%-SF-M-CuO composite film represents a promising candidate for active food packaging applications in meat, vegetable, and fruit products. Further investigations using real food systems are required to confirm its practical antimicrobial performance.

## Figures and Tables

**Table 1:** EDX elemental composition analysis of composite films.

Samples	Element (Wt.%)					
	C	O	Cu	Ag	Mg	Si
PLA	82.82	17.18	-	-	-	-
PLA-4%-CCM	81.83	18.17	-	-	-	-
PLA-4%-CCM-1%-SF-M-CuO	79.19	18.23	1.11	1.47	ND	ND
PLA-4%-CCM-2%-SF-M-CuO	81.34	16.48	2.12	0.06	ND	ND
PLA-4%-CCM-3%-SF-M-CuO	76.49	16.02	7.24	0.25	ND	ND
PLA-4%-CCM-4%-SF-M-CuO	62.55	15.25	20.02	2.15	0.04	ND

ND—Not detectable.

**Table 2:** The antimicrobial activity of composite films.

Samples	*S. aureus*			*E. coli*		
	Viable Bacteria (CFU/mL)	R (%)	Log10 Reduction	Viable Bacteria (CFU/mL)	R (%)	Log10 Reduction
PLA	1.20 ± 0.07 × 10^10^	0	-	7.33 ± 0.58 × 10^8^	0	-
PLA-4%-CCM	1.80 ± 0.10 × 10^8^	98.50	1.83	2.60 ± 0.20 × 10^7^	96.45	1.45
PLA-4%-CCM-1%-SF-M-CuO	1.00 ± 0.00 × 10^4^	99.99	6.08	8.00 ± 2.00 × 10^4^	99.99	3.95
PLA-4%-CCM-2%-SF-M-CuO	0.67 ± 0.00 × 10^2^	99.99	8.22	2.07 ± 0.20 × 10^3^	99.99	5.55
PLA-4%-CCM-3%-SF-M-CuO	Below LOD	99.99	>8.22 *	Below LOD	99.99	>5.55 *
PLA-4%-CCM-4%-SF-M-CuO	Below LOD	99.99	>8.22 *	Below LOD	99.99	>5.55 *

**R**: Percentage reduction in bacterial growth. Values are expressed as mean ± SD (*n* = 3). Statistical differences were evaluated using one-way ANOVA followed by Tukey’s post hoc test (*p* < 0.05). **Below LOD** indicates bacterial counts below the limit of detection (<100 CFU/mL). * Log reduction values marked with an asterisk were estimated using the LOD because no bacterial colonies were detected.

## Data Availability

Data will be made available on request.

## Supplementary Materials

The following supporting information can be downloaded at: https://www.mdpi.com/article/10.3390/polym18131626/s1, Table S1: EDX elemental composition of synthesized dual-metal-doped copper oxide nanoparticles; Figure S1: XRD analysis of curcumin powder sample; Figure S2: EDX analysis of composite films.

## References

[1] Phan A., Mijar S., Harvey C., Biswas D. (2025). *Staphylococcus aureus* in Foodborne Diseases and Alternative Intervention Strategies to Overcome Antibiotic Resistance by Using Natural Antimicrobials. Microorganisms.

[2] Cao J., Cui W., Luo L., Xie G. (2026). Collaborative Supervision for Sustainable Governance of the Prepared Food Industry in China: An Evolutionary Game and Markov Chain Approach. Sustainability.

[3] Silvee S.S., Silvee S.S. (2026). Introduction to the Law of Food Security: A Legal Framework for Global Food Justice and Sustainability. The Law of Food Security: The Legal Framework for a Sustainable and Equitable Global Food System.

[4] Mithuna R., Tharanyalakshmi R., Jain I., Singhal S., Sikarwar D., Das S., Ranjitha J., Ghosh D., Rahman M.M., Das B. (2024). Emergence of Antibiotic Resistance Due to the Excessive Use of Antibiotics in Medicines and Feed Additives: A global scenario with emphasis on the Indian perspective. Emerg. Contam..

[5] Tian L., Khalil S., Bayen S. (2017). Effect of Thermal Treatments on the Degradation of Antibiotic Residues in Food. Crit. Rev. Food Sci. Nutr..

[6] Jiang H., Liu R., Huang X., Qin L. (2026). Effect of Various Thermal Treatments on Erythromycin Residues and Degradation Products in Turbot Fish Meat: Implications for Food Safety. Foods.

[7] Thamaraiselvi C., Athira S.T., Vasanthy M., Jesudass J.S., Gnanajothi K., Chandrasekaran R. (2026). Sustainable Management of Coffee Cherry Pulping Wastewater Using Biogenically Synthesized Calcium Oxide Nanoparticles. Next Mater..

[8] Zolfaghari M., Yadegar A., Rezaei A., Rafieian F., Kazemi M., Fazeli H., Karbasizade V. (2026). Plant-mediated green synthesis of zinc oxide nanoparticles using *Anvillea garcinii* extract: Characterization and investigation of their anticancer, antibacterial and antioxidant effects. Ind. Crops Prod..

[9] Alhamdany S.R., Alsalhy Q.F., Hassan A.K., Al-Saadi S., Meskher H., Al-Juboori R.A. (2026). Eco-friendly Synthesis of Metal Oxide Nanoparticles (ZnO, MgO and CuO) for Efficient Water Purification: A Review. Mater. Today Commun..

[10] Abd I.K., Ismail R.K., Hussan S.M.A.A., Al-Jashamy K., Salman A.D., Abd A.K. (2026). Green Synthesis of Copper Oxide Nanoparticles Using Fig Leaves Extract and Their Use as an Antibacterial Agent. Next Res..

[11] Wang H., Liu Y., Yi Y., Min T., Wang H., Ke L., Hu W., Hang C., Song Z., Zhou M. (2026). Advances in Active Packaging for Enhanced Food Safety: Insights into Functional Additives, Response Strategies and Applications. Food Control.

[12] Westlake J.R., Tran M.W., Jiang Y., Zhang X., Burrows A.D., Xie M. (2022). Biodegradable Active Packaging with Controlled Release: Principles, Progress, and Prospects. ACS Food Sci. Technol..

[13] Bint-E-Zafar A., Ahsan S., Chughtai M.F.J., Khaliq A., Saima H., Mehmood T., Liaqat A., Farooq M.A., Sameed N., Amir R.M. (2025). Sustainable Edible Packaging: Advances in Materials, Manufacturing, and Applications. eFood.

[14] Oyewole O.A., Amole O.F., Majin E.N., Maddela N.R. (2026). Antimicrobial Nanomaterials in Food Packaging and Preservation. RSC Adv..

[15] Pérez E., Sanjuán E., Jůzl M., Raposo A., Saraiva A., Jaber J.R., Carrascosa C. (2026). Active Antimicrobial Packaging Systems: Mechanisms of Microbial Control and Applications in Food Preservation. Biology.

[16] Shao B., Huo Y., Yang Q., Zhao F., Ju J. (2026). Beyond Tradition: Optimization Strategy for Mechanical Properties of New Generation Biodegradable Packaging Materials. Compr. Rev. Food Sci. Food Saf..

[17] Swetha T.A., Bora A., Mohanrasu K., Balaji P., Raja R., Ponnuchamy K., Muthusamy G., Arun A. (2023). A Comprehensive Review on Polylactic Acid (PLA)—Synthesis, Processing and Application in Food Packaging. Int. J. Biol. Macromol..

[18] Islam M.S., Elahee G.M.F., Fang Y., Yu X., Advincula R.C., Cao C. (2025). Polylactic Acid (PLA)-Based Multifunctional and Biodegradable Nanocomposites and Their Applications. Compos. B Eng..

[19] Rajendran D.S., Venkataraman S., Jha S.K., Chakrabarty D., Kumar V.V. (2024). A Review on Bio-Based Polymer Polylactic Acid Potential on Sustainable Food Packaging. Food Sci. Biotechnol..

[20] Zende R., Ghase V., Jamdar V. (2025). Recent Advances in the Antimicrobial and Antioxidant Capabilities of PLA Based Active Food Packaging. Polym.-Plast. Technol. Mater..

[21] Gutiérrez-Ruiz S.C., Romero-Montero A., Hernández-Parra H., Peña-Corona S.I., García-Gasca H.A., Carbone K., Gervasi F., Mangalpady S.S., Kaverikana R., Shanaida M. (2026). Curcumin Nanoformulations for Dermatological Applications: From Nutraceuticals to Nanocarriers. Food Front..

[22] Hewlings S.J., Kalman D.S. (2017). Curcumin: A Review of Its Effects on Human Health. Foods.

[23] Roy S., Rhim J.-W. (2020). Preparation of Bioactive Functional Poly(Lactic Acid)/Curcumin Composite Film For Food Packaging Application. Int. J. Biol. Macromol..

[24] Roy S., Rhim J.-W. (2020). Preparation of Antimicrobial and Antioxidant Gelatin/Curcumin Composite Films for Active Food Packaging Application. Colloids Surf. B Biointerfaces.

[25] Zhou S., Li N., Peng H., Yang X., Lin D. (2023). The Development of Highly pH-Sensitive Bacterial Cellulose Nanofibers/Gelatin-Based Intelligent Films Loaded with Anthocyanin/Curcumin for the Fresh-Keeping and Freshness Detection of Fresh Pork. Foods.

[26] Rathod N.V., Dalai P., Agrawal-Rajput R., Mishra S. (2025). Design, Synthesis and Molecular Modelling Studies of Bile Acid-Curcumin Conjugates as Potential Antiproliferative Agents for Breast Cancer. Bioorg. Med. Chem..

[27] Gopinath K., Gnanasekar S., Al-Ghanim K.A., Nicoletti M., Govindarajan M., Arumugam A., Balalakshmi C., Thanakkasaranee S. (2023). Fabrication of Neodymium (Nd), Cadmium (Cd) and Nd:Cd Doped Hybrid Copper Oxide Nanocomposites: Evaluation of Their Antibacterial Activity and Cytotoxicity Against Human L132 Cell Line. Ceram. Int..

[28] Gopinath K., Sathishkumar G., Xu L. (2024). An Overview of the Copper Oxide Nanofillers Integrated in Food Packaging Systems. Coatings.

[29] Kasi G., Thanakkasaranee S., Stalin N., Arumugam A., Jantanasakulwong K., Panyathip R., Sukunta J., Tanadchangsaeng N., Worajittiphon P., Rachtanapun P. (2024). Enhancement of Antimicrobial Properties and Cytocompatibility Through Silver and Magnesium Doping Strategies on Copper Oxide Nanocomposites. J. Alloys Compd..

[30] Cheng J., Lin X., Wu X., Liu Q., Wan S., Zhang Y. (2021). Preparation of A Multifunctional Silver Nanoparticles Polylactic Acid Food Packaging Film Using Mango Peel Extract. Int. J. Biol. Macromol..

[31] Kim I., Viswanathan K., Kasi G., Sadeghi K., Thanakkasaranee S., Seo J. (2019). Poly(Lactic Acid)/ZnO Bionanocomposite Films with Positively Charged ZnO as Potential Antimicrobial Food Packaging Materials. Polymers.

[32] Kim D., Jang M., Seo J., Nam K.-H., Han H., Khan S.B. (2013). UV-cured poly(urethane acrylate) Composite Films Containing Surface-Modified Tetrapod ZnO Whiskers. Compos. Sci. Technol..

[33] Palaniyappan S., Sivakumar N.K., Bodaghi M., Rahaman M., Pandiaraj S. (2024). Preparation and Performance Evaluation of 3D Printed Poly Lactic Acid Composites Reinforced with Silane Functionalized Walnut Shell for Food Packaging Applications. Food Packag. Shelf Life.

[34] Chennaiah M.B., Abraar S.A.M., Arun M., Vardhan T.V., Velusamy K., Kumar A.M., Gurumoothy S., Elsheikh A.H., Ramesh B. (2024). Development of Biocomposite Food Packaging Coating Material with Silane-treated Nanosilica and Grape Seed Oil Blended Vinyl Ester. Silicon.

[35] Mousavi M., Fini E. (2020). Silanization Mechanism of Silica Nanoparticles in Bitumen Using 3-Aminopropyl Triethoxysilane (APTES) and 3-Glycidyloxypropyl Trimethoxysilane (GPTMS). ACS Sustain. Chem. Eng..

[36] Maluin F.N., Katas H. (2022). Chitosan Functionalization of Metal- and Carbon-Based Nanomaterials as an Approach Toward Sustainability Tomorrow. Nanotoxicology.

[37] Farouk A., Moussa S., Ulbricht M., Schollmeyer E., Textor T. (2014). ZnO-Modified Hybrid Polymers as an Antibacterial Finish for Textiles. Text. Res. J..

[38] Buşilă M., Muşat V., Textor T., Mahltig B. (2015). Synthesis and Characterization of Antimicrobial Textile Finishing Based on Ag:ZnO Nanoparticles/Chitosan Biocomposites. RSC Adv..

[39] Lallo da Silva B., Caetano B.L., Chiari-Andréo B.G., Pietro R.C.L.R., Chiavacci L.A. (2019). Increased Antibacterial Activity of ZnO Nanoparticles: Influence of Size and Surface Modification. Colloids Surf. B Biointerfaces.

[40] Zhang Z., Liu S., Xiong H., Jing X., Xie Z., Chen X., Huang Y. (2015). Electrospun PLA/MWCNTs Composite Nanofibers for Combined Chemo- and Photothermal Therapy. Acta Biomater..

[41] Meng F., Yan X., Nkede F.N., Wardak M.H., Van T.T., Tanaka F., Tanaka F. (2024). An Intelligent Chitosan/Polyvinyl Alcohol Film with Two Types of Anthocyanins for Improved Color Recognition Accuracy and Monitoring Fresh-Cut Pineapple Freshness. Food Packag. Shelf Life.

[42] Eze F.N., Jayeoye T.J., Eze R.C., Ovatlarnporn C. (2024). Construction of Carboxymethyl Chitosan/PVA/Chitin Nanowhiskers Multicomponent Film Activated with *Cotylelobium lanceolatum* Phenolics and In Situ SeNP for Enhanced Packaging Application. Int. J. Biol. Macromol..

[43] Pacharra S., McMahon S., Duffy P., Basnett P., Yu W., Seisel S., Stervbo U., Babel N., Roy I., Viebahn R. (2020). Cytocompatibility Evaluation of a Novel Series of PEG-Functionalized Lactide-Caprolactone Copolymer Biomaterials for Cardiovascular Applications. Front. Bioeng. Biotechnol..

[44] Huang T., He X., Ali A., Gnanasekar S., Xiang Y., Zhang K., Rao X., Kang E.-T., Xu L.Q. (2024). Phytic Acid-Promoted Deposition of Gold Nanoparticles with Grafted Cationic Polymer Brushes for the Construction of Synergistic Contact-Killing and Photothermal Bactericidal Coatings. ACS Appl. Bio Mater..

[45] Kogje M., Mestry S., Mohanty J.D., Mhaske S.T. (2025). Modification of Fly Ash Cenospheres by 3-Glycidyloxypropyl Trimethoxysilane (GPTMS) for Anticorrosive Coating Applications. Iran. Polym. J..

[46] Li C.-P., Weng M.-C., Huang S.-L. (2020). Preparation and Characterization of pH Sensitive Chitosan/3-Glycidyloxypropyl Trimethoxysilane (GPTMS) Hydrogels by Sol-Gel Method. Polymers.

[47] Saliba P.A., Mansur A.A., Santos D.B., Mansur H.S. (2015). Fusion-Bonded Epoxy Composite Coatings on Chemically Functionalized API Steel Surfaces for Potential Deep-Water Petroleum Exploration. Appl. Adhes. Sci..

[48] Kim H.-L., Park S.-Y., Rhim J.-W., Lee H.-J., Her J.-Y. (2025). Fabrication and Characterization of PLA-Based Composite Films Incorporated with Copper Oxide Nanoparticles and Lavender Essential Oil. Int. J. Polym. Sci..

[49] Chopra S., Pande K., Puranam P., Deshmukh A.D., Bhone A., Kale R., Galande A., Mehtre B., Tagad J., Tidake S. (2023). Explication of Mechanism Governing Atmospheric Degradation of 3D-printed Poly(lactic acid) (PLA) with Different In-Fill Pattern And Varying In-Fill Density. RSC Adv..

[50] Weng Q.H., Hu M.H., Wang J.F., Hu J.J. (2025). Enhancing the Flexibility and Hydrophilicity of PLA via Polymer Blends: Electrospinning vs. Solvent Casting. Polymers.

[51] Jamshidian M., Tehrany E.A., Imran M., Jacquot M., Desobry S. (2010). Poly-Lactic Acid: Production, Applications, Nanocomposites, and Release Studies. Compr. Rev. Food Sci. Food Saf..

[52] Wijayawardana S., Thambiliyagodage C., Jayanetti M. (2024). Kinetic Study of In Vitro Release of Curcumin From Chitosan Biopolymer and The Evaluation of Biological Efficacy. Arab. J. Chem..

[53] Yang J.-Y., Kim D.-K., Han W., Park J.-Y., Kim K.-W., Kim B.-J. (2022). Effect of Nucleating Agents Addition on Thermal and Mechanical Properties of Natural Fiber-Reinforced Polylactic Acid Composites. Polymers.

[54] Mondal K., Soundararajan N., Goud V.V., Katiyar V. (2024). Cellulose Nanocrystals Modulate Curcumin Migration in PLA-Based Active Films and Its Application as Secondary Packaging. ACS Sustain. Chem. Eng..

[55] Hasanin M.S., Youssef A.M. (2022). Ecofriendly Bioactive Film Doped CuO Nanoparticles Based Biopolymers and Reinforced by Enzymatically Modified Nanocellulose Fibers for Active Packaging Applications. Food Packag. Shelf Life.

[56] Ragab H.M., Diab N.S., Aziz R.A., Elneim E.A.A., Alghamdi A.M., Tarabiah A.E., Farea M.O. (2025). Effects of CuO Nanoparticles on The Physical and Functional Properties of Biodegradable Polymer-Based Composites for Biomedical and Flexible Packaging Applications. J. Vinyl Addit. Technol..

[57] Gunaki M.N., Masti S.P., D’Souza O.J., Eelager M.P., Kurabetta L.K., Chougale R.B., Kadapure A.J., Praveen Kumar S.K. (2024). Fabrication of CuO Nanoparticles Embedded Novel Chitosan/Hydroxypropyl Cellulose Bio-Nanocomposites for Active Packaging of Jamun Fruit. Food Hydrocoll..

[58] Shawono B.G., Etefa K.T., Muleta G.G., Kai Z., Beyene T.T. (2025). Eco-friendly Synthesis of Ag-doped CuO Nanocomposites Using *Solanum tuberosum* Peel Extract, Showcasing Enhanced Antimicrobial, Antioxidant, and Photocatalytic Performance. Inorg. Chem. Commun..

[59] Das D., Paul P., Mandal P. (2025). Prolonging Mulberry Leaf Longevity with Silver-Doped Copper Oxide Nanoparticles: A Postharvest Approach. Discov. Plants.

[60] Jiao Z.-H., Li M., Feng Y.-X., Shi J.-C., Zhang J., Shao B. (2014). Hormesis Effects of Silver Nanoparticles at Non-Cytotoxic Doses to Human Hepatoma Cells. PLoS ONE.

[61] Patel M.K., Zafaryab M., Rizvi M., Agrawal V.V., Ansari Z., Malhotra B., Ansari S. (2013). Antibacterial and Cytotoxic Effect of Magnesium Oxide Nanoparticles on Bacterial and Human Cells. J. Nanoeng. Nanomanuf..

[62] Osman H., Tang X., Liu Y., Zou G., Jiang T., Wang Y., Wang T., Bai X. (2025). Polylactic Acid Based Composite Fibers for Synergistic Photothermal and Photodynamic Antibacterial Effect with Enhanced Bone Regeneration. Mater. Today Chem..

[63] Xu N., Zhang X., Qi T., Wu Y., Xie X., Chen F., Shao D., Liao J. (2022). Biomedical Applications and Prospects of Temperature-Orchestrated Photothermal Therapy. MedComm–Biomater. Appl..

[64] Qi F., He L., Cui L., Wang W., Siddique K.H.M., Li S. (2023). Smart Antibacterial Food Packaging Based on MIL-53 (Fe) Functionalized Polylactic Acid Film for pH-Responsive Controlled Release. J. Polym. Environ..

[65] Zahidova F., Yildiz S., Özdemir A., Gülfen M., Yemiş G.P. (2023). Modification of Poly(L-Lactic Acid)-Based Films and Evaluation of Physical and Antibacterial Properties by Using Multivariate Data Analysis. Int. J. Biol. Macromol..

[66] Gao C., Chen P., Ma Y., Sun L., Yan Y., Ding Y., Sun L. (2023). Multifunctional Polylactic Acid Biocomposite Film for Active Food Packaging with UV Resistance, Antioxidant and Antibacterial Properties. Int. J. Biol. Macromol..

[67] Yaman M., Yildiz S., Özdemir A., Yemiş G.P. (2024). Multicomponent System for Development of Antimicrobial PLA-based Films with Enhanced Physical Characteristics. Int. J. Biol. Macromol..

[68] Akshaykranth A., Jayarambabu N., Kumar A., Venkatappa Rao T., Kumar R.R., Srinivasa Rao L. (2022). Novel Nanocomposite Polylactic Acid Films with Curcumin-ZnO: Structural, Thermal, Optical and Antibacterial Properties. Curr. Res. Green Sustain. Chem..

