# Supplementary material for: Development of Polylactic Acid–Curcumin Composite Films with Dual-Metal-Doped Copper Oxide Nanoparticles for Sustainable Antioxidant, Biocompatible, Photothermal, and Antibacterial Performance
Source: Polymers (Basel). 2026 Jun 30;18(13):1626. doi: 10.3390/polym18131626 (PMC13364498; doi:10.3390/polym18131626)
Supplement: Supplementary file 1 [file polymers-18-01626-s001.zip › polymers-4354886-supplementary.pdf]

Supplementary Material

# Development of polylactic acid–curcumin composite films with dual-metal-doped copper oxide nanoparticles for sustainable antioxidant, biocompatible, photothermal, and antibacterial performance

**Gopinath Kasi [1], Sarinthip Thanakkasaranee [1,2,3], Nattan Stalin [4], Tae-Sik Park [4], Ramar Dharmaraj [5], Kittisak Jantanasakulwong [1,2,3], Nuttapol Tanadchangsaeng [6], Pornchai Rachtanapun [1,2,3,*]**

[1] Division of Packaging Technology, Faculty of Agro-Industry, Chiang Mai University, 50100, Thailand; gopiscientist@gmail.com (G.K.); sarinthip.t@cmu.ac.th (S.T); kittisak.jan@cmu.ac.th (K.J.)
[2] Center of Excellence in Agro Bio-Circular-Green Industry (Agro BCG), Chiang Mai University, Chiang Mai 50100, Thailand.
[3] Center of Excellence in Materials Science and Technology, Chiang Mai University, Chiang Mai 50200, Thailand.
[4] Department of Life Science, Gachon University, Seongnam, Gyeonggi-do 13120, Republic of Korea; nattanstalin@gmail.com (N.S); tspark@gachon.ac.kr (SP)
[5] Department of Botany, Alagappa University, Karaikudi, 630 003, Tamil Nadu, India; dharmarajramar1025@gmail.com (R.D)
[6] College of Biomedical Engineering, Rangsit University, Lak-Hok, Pathumthani, Thailand; nuttapol.t@rsu.ac.th (N.T)
* Correspondence: pornchai.r@cmu.ac.th; Tel.: +66-635-492-556

**Table S1.** EDX elemental composition of synthesized dual-metal-doped copper oxide nanoparticles.

| **Element** | **Wt.%** | **At.%** |
|---|---|---|
| Ag | 1.90 | 0.65 |
| Mg | 2.73 | 4.15 |
| Cu | 72.44 | 42.17 |
| O | 22.94 | 53.03 |
| Total | 100.00 | 100.00 |

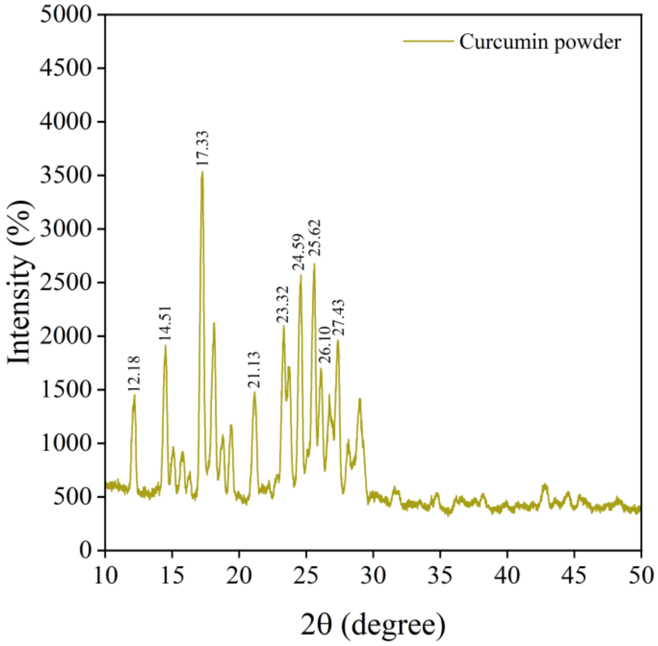


**Figure S1.** XRD analysis of curcumin powder sample.

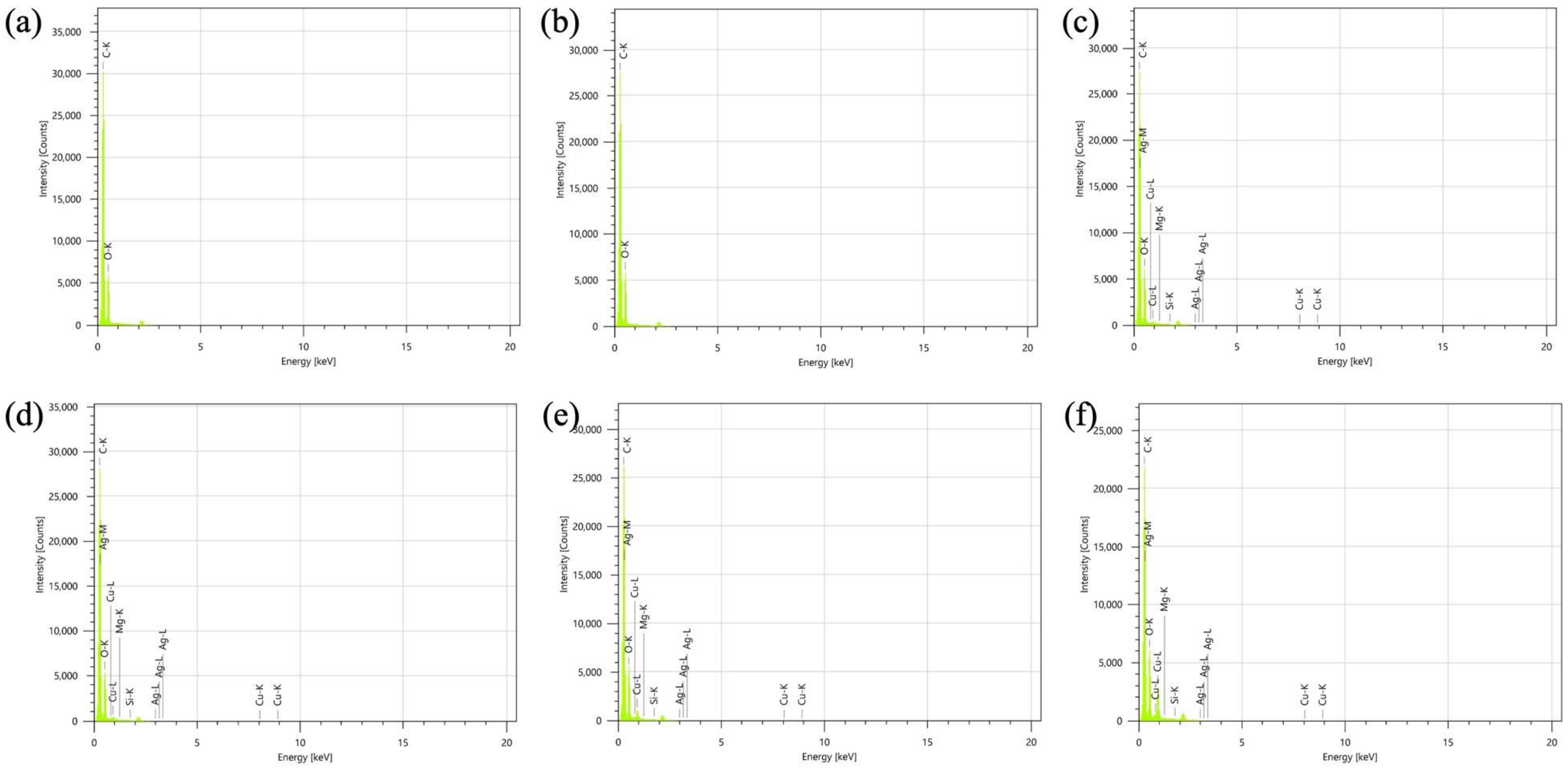


**Figure S2.** EDX analysis of composite films: (**a**) PLA; (**b**) PLA-4%-CCM; (**c**) PLA-4%-CCM-1%-SF-M-CuO; (**d**) PLA-4%-CCM-2%-SF-M-CuO; (**e**) PLA-4%-CCM-3%-SF-M-CuO, and (**f**) PLA-4%-CCM-4%-SF-M-CuO.
